# Anterior Cruciate Ligament Reconstruction With the All-Inside Technique: Equivalent Outcomes and Failure Rate at Three-Year Follow-Up Compared to a Doubled Semitendinosus-Gracilis Graft

**DOI:** 10.7759/cureus.20508

**Published:** 2021-12-18

**Authors:** George Kyriakopoulos, Spyros Manthas, Maria Vlachou, Leon Oikonomou, Stamatios A Papadakis, Konstantinos Kateros

**Affiliations:** 1 A' Orthopaedic Department, General Hospital G. Gennimatas, Athens, GRC; 2 Department of Trauma and Orthopaedics, General Hospital of Livadeia, Livadeia, GRC; 3 A' Orthopaedic Department, General Hospital G.Gennimatas, Athens, GRC; 4 B' Department of Orhopaedics, KAT General Hospital of Attica, Kifisia, GRC

**Keywords:** acl, tibial interference screw, suspensory fixation, semitendinosus and gracilis graft, quadrupled semitendinosus, all-inside, anterior cruciate ligament (acl) reconstruction

## Abstract

Purpose: To compare in terms of failure rates, clinical and functional outcomes the all-inside anterior cruciate ligament (ACL) reconstruction with double suspensory fixation and quadrupled semitendinosus autograft with anteromedial portal doubled semitendinosus-gracilis autograft with suspensory femoral and tibial interference screw fixation.

Methods: Forty-four patients were sequentially allocated into two groups and followed up prospectively for a 3-year period. The first group was the all-inside group and the second was the “classic” AM portal with S-G graft. Each group comprised 22 patients. All patients underwent KT-1000 testing preoperatively and at a minimum of six months postoperatively. Visual pain analog was recorded preoperatively and during both the immediate postoperative period and throughout the follow-up. The Lysholm knee score was used comparing the preoperative and 24-month timepoints.

Results: The visual analog scale (VAS) pain scores showed a significant difference at two weeks (2.4 vs 1.8, p < 0.01 ) in favor of the all-inside group, but that difference disappeared in the later follow-up visits. Similarly, there was no significant difference with Lysholm knee scores at two years and the side-to-side anterior translation measured with the KT-1000. At the three-year mark, there were no failures in either of the groups.

Conclusion: The all-inside technique appears to be equivalent in terms of outcomes to the classic S-G technique, and given the less-invasive nature and versatility in graft choices is a safe and effective technique for primary ACL reconstruction.

## Introduction

Anterior cruciate ligament (ACL) injury is a common injury, especially among athletic individuals, with ACL reconstructions being well over 100,000 per year in the United States alone [1-7]. In recent years there has been an emerging interest in less invasive techniques and their potential benefits both in terms of morbidity and facility of possible future revision surgery.

The all-inside technique has been described over 20 years ago and was recently popularized by Lubowich et al. [8,9]. The technique entails short tunnels and the use of quadrupled semitendinosus graft with dual suspensory fixation.

As with all novel techniques, the benefits of reduced donor site morbidity and less invasive nature, have been met with some skepticism, mainly focused on the potential inferiority of the short graft and suspensory fixation both in terms of graft failures and clinical outcome equivalence.

The aim of the present study is to compare the all-inside technique with the use of quadrupled semitendinosus graft to the standard tibial tunnel screw fixation with a semitendinosus-gracilis graft in terms of both objective and subjective patient outcomes.

## Materials and methods

Inclusion criteria

All patients who were going to undergo ACL reconstruction during a 12-month period by the senior authors were included in the current study. Only skeletally mature patients were included. Patients with previous ACL surgery or multiligamentous instability were excluded.

In total, 44 patients were included and sequentially allocated to either the standard or all-inside group.

The study was approved by the General Hospital of Athens Ethical and Scientific Committee (approval no 34813271119).

All patients underwent physiotherapy preoperatively to restore range of motion and muscle strength. Preoperatively Lysholm, Visual analog pain scores and KT-1000 mechanical testing were performed. 

The classic group underwent hamstring ACL reconstruction using both gracilis and semitendinosus tendons harvested through an anteromedial incision. (A 5-cm incision was performed over the anteromedial aspect of the tibia, beginning 5 cm distal to the joint line and 2.5 cm medial to the tibial tuberosity. Once identified, the aponeurosis was opened in line with its distal fibers, and the semitendinosus and gracilis tendon were harvested) The femoral tunnel was reamed antegrade through an anteromedial portal and the graft was stabilized with suspensory button fixation at the femur and a bioabsorbable screw at the tibial tunnel. 

In the all-inside group, a single semitendinosus tendon was harvested from an anteromedial incision. (A 5-cm incision was performed over the anteromedial aspect of the tibia, beginning 5 cm distal to the joint line and 2.5 cm medial to the tibial tuberosity. Once identified, the aponeurosis was opened in line with its distal fibers, and the semitendinosus was separated from the gracilis tendon). The femoral tunnel was drilled antegrade and reamed to 20-30 mm depth depending on graft length. The tibial tunnel was originally drilled antegrade and reamed retrograde with a flip-cutter. The graft was stabilized with suspensory fixation both in the femur and the tibia.

A tourniquet was used in all patients and a drain was placed, that was removed on the first postoperative day.

Postoperatively no brace was used and the patients were instructed to weight-bear as tolerated on their heels. Thromboprophylaxis was used as per national protocols and simple range of motion and strengthening exercises were instructed for the first two weeks. After suture removal at the two-week follow-up, formal physiotherapy was commenced coordinated by the same physiotherapist. The patients were followed up at two weeks postoperatively, six weeks, three months, six months and every six months after that.

A visual analog scale (VAS) pain score was obtained at each follow-up visit, KT-1000 testing was performed at a minimum of six months postoperatively and the Lysholm score at the 24-month follow-up visit. The patients were followed up for another 12 months with a final visit at 36 months.

The KT-1000 testing was performed with the knee in 20-30 degrees of flexion with the application of a posterior-anterior directed force of 138 Newtons and posteroanterior translation was measured in both knees. To account for possible variability patients were examined three times after low-impact aerobic exercise and the largest translation values were used.

## Results

A total of 44 patients, 22 in each group participated in the study. There was a male predominance with 34 male and 10 female patients and the right knee was the most common side with 30 vs 14 left knees. The groups were demographically matched, with no significant differences in age and sex composition (Table 1).

**Table 1:** Sample demographics No significant difference between groups is observed

Group	All-inside	Classic
Age average (range)	27.5 (19-40)	26.6 (18-37)
Female/male ratio	5/17	4/18
Female percentage	30%	22%

Statistical analysis was carried out using the statistical package SPSS version 21.00 (IBM Corporation, Somers, NY, USA). All tests were two-sided, statistical significance was set at p < 0.05.

Preoperative versus postoperative Lysholm scores between groups as well as the percentile change were compared using independent samples t-test. Preoperative versus postoperative values for each team were performed with a paired samples t-test.

VAS pain scores between groups for every time-point were performed with a two-way ANOVA model, whereas comparison between different time-points in each group with one-way repeated measures ANOVA. In KT-1000 results, the difference of posteroanterior translation between injured and healthy limb pre- and postoperatively between groups was performed with independent samples t-test. Comparison of preoperative versus postoperative values for each group was performed using paired samples t-test. The adjusted absolute difference between pre and postoperative values between groups was done using analysis of covariance model (ANCOVA).

The minimum follow-up was set at 36 months.

**Figure 1:**
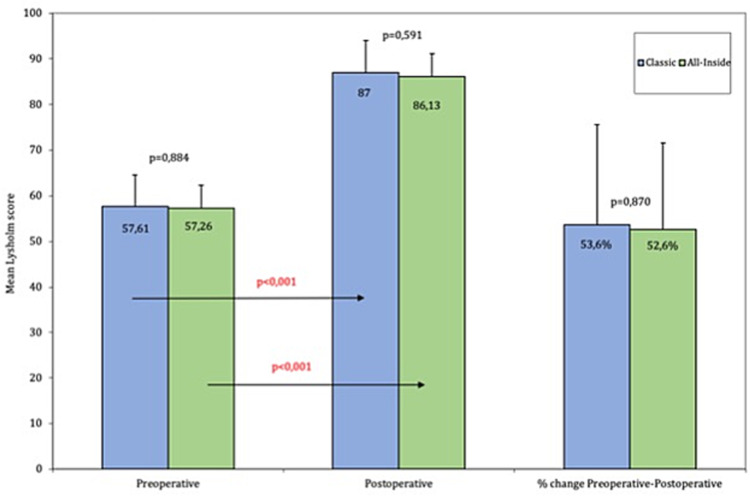
Lysholm Knee Score The scores from the classic S-G tibial interference screw are in blue and the all-inside group is in green. The mean preoperative and 24-month follow-up values, as well as the mean percentile change, are drawn. The statistical significance of the difference between groups is above the corresponding columns and the intra-group difference above the corresponding arrows.

In terms of subjective outcomes, the Lysholm knee scores showed a significant improvement compared to the preoperative values in both groups. The preoperative values were almost identical in both groups with a mean of 57.6 and 57.2 points in the classic and the all-inside groups respectively. The 24-month postoperative scores for the classic group increased significantly to an average of 87 (p < 0.001), and similar results were found in the all-inside groups, with a mean Lysholm score of 86.13 (p < 0.001). There was no significant difference between groups either in terms of postoperative values or percentile change (Figure 1).

**Figure 2:**
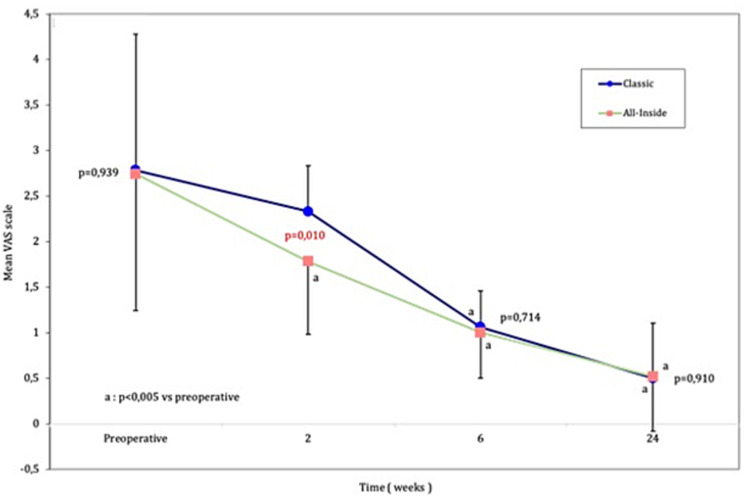
Visual Analog Pain scores The classic S-G group is in blue and the all-inside group in green. The difference between pre- and postoperative values is marked with an a, where it reached statistical significance. The difference between groups is shown at each time point.

With regards to reported pain level, as described by the VAS pain scores both groups demonstrated a decrease in self-reported pain levels that were progressive in nature throughout the two-, six-, and 24-week time point, after which no significant change was observed. In the early postoperative period (two-week mark) the all-inside group had significantly (p < 0.01) lower pain scores compared to the classic group. This advantage was, however, short-lived as the six- and 24-week pain scores did not differ significantly between groups. The less invasive nature of the technique is probably the reason for this difference. Both groups showed a significant (p < 0.005) decrease in pain levels between the preoperative and the six- and 24-week time-points, with the all-inside groups showing a significant decrease in pain in all postoperative time points (Figure 2).

**Figure 3:**
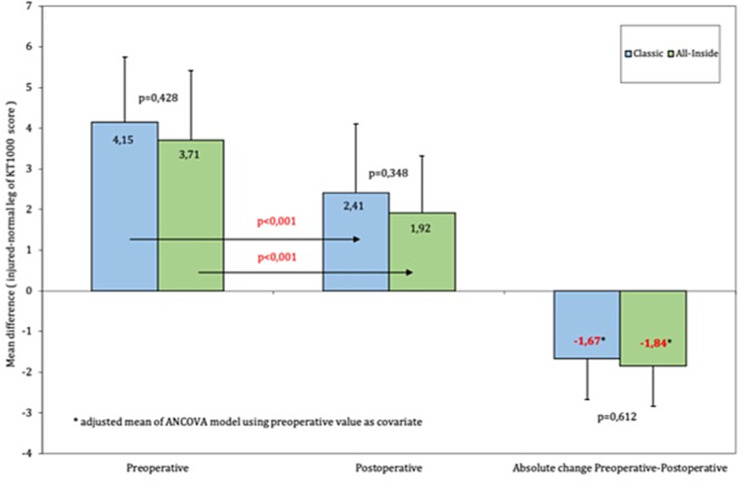
KT-1000 testing In blue are the scores of the classic S-G group and in green are the scores of the all-inside group. Both groups significantly improved in terms of posteroanterior translation compared to preoperative values.

The objective posteroanterior laxity measurements were conducted using the KT-1000 at a minimum of six months postoperatively (range 6-9 months). Both groups had an average side-to-side difference of over 3 mm, with the classic group patients showing a mean difference of 4.15 and the all-inside group 3.71 mm, a difference that did not reach statistical significance, however. Postoperatively both groups had a significant reduction in AP laxity (p < 0.001) with a mean side-to-side difference of 2.41 mm for the classic group and 1.92 for the all-inside group. The absolute reduction in PA translation did not differ significantly between groups and neither did the postoperative values (Figure 3).

At the final follow-up visit at 36 months, we observed no graft failure requiring revision ACL. One patient, however, in the classic group did show excessive (6 mm) side-to-side difference in the KT-1000 test at one year, but they elected not to undergo revision. One patient in the all-inside group developed a postoperative hematoma, for which they underwent arthroscopic washout and implant retention, and which resolved uneventfully. No infections were noted or other serious adverse events.

## Discussion

The all-inside technique is a relatively new technique that is less invasive compared to classic tendon harvesting and fixation techniques both in terms of donor site harvesting and bony preparation. There have been however concerns regarding various possible modes of premature failure. 

The biology of fixation has been questioned both in terms of tunnel length and tendon to bone vs bone to bone healing. Short sockets have been shown to have superior tendon to bone fixation compared to standard long tunnels in a histologic canine study by Smith et al. [10]. Human data have shown similar incorporation in an MRI study comparing quadrupled semitendinosus tendon with short tunnels and semitendinosus-gracilis tendon with standard tunnels. The authors used tibial screw and suspensory button femoral fixation in both groups [3]. Lubowic et al. have demonstrated equivalent clinical outcomes between patients with short or standard tibial tunnels using allograft tibialis posterior tendons in a prospective randomized study [11]. It seems that tendon to bone attachment occurs via the Sharpey fibers and takes place in the vicinity of the joint, rendering tunnel lengths over 15 mm biologically irrelevant [4,12,13].

Bone-tendon grafts such as the patellar bone tendon graft have been long thought to have improved incorporation and have been also the golden standard in revision ACL surgery. However, in primary ACL reconstruction, clinical data at long-term follow up as shown by Gifstad et al. in a study comparing bone-patellar tendon-bone (BPTB) with semitendinosus-gracilis autograft with a minimum seven-year follow up are similar [14]. Even the more demanding subgroups, such as the younger more active patients have been shown to have similar outcomes in a randomized controlled trial. The authors compared clinical outcomes and KT-1000 mechanical testing in highly active patients under 24 years of age finding no difference at two years of follow-up [15].

Our findings are similar with equivalent mechanical testing and clinical outcomes at the one-year follow-up and similar failure rates at two years between the all-inside and the full tibial tunnel group.

Another point of concern has been the fixation durability with criticism both in terms of the choice of button suspensory fixation over the screw, and the possibility of graft lengthening and tunnel widening via the wind sweeper effect. Such results were reported by Bressy et al. [2] who followed 35 patients with all-inside ACL reconstruction and found that only 54% had an anterior drawer of less than 3 mm at 12 months. We have used the same system and found under 5% side to side difference over 3 mm at an average of seven months (6-9), which seems more consistent with the current literature [6].

Button fixation, especially on the femoral side, has been well established and has shown equivalent long-term outcomes with screw fixation with both hamstring and patellar tendon grafts in one of the longest follow-up studies in the literature [16]. Lubowitz et al. report similar results comparing both side screw vs suspensory button fixation at a shorter follow-up, in their prospective RCT [17]. Likewise, Yasen et al. [18] report a 6.5% failure rate at the three-year to follow up which they attribute mostly to violent episodes/re-injury.

Graft lengthening, especially using the adjustable loop buttons has been a concern. Mayr et al. [19] conducted a cadaveric biomechanical study with an array of graft configurations with non-pretensioned graft and found significant graft lengthening in all specimens. Graft pretensioning was shown by Monaco et al. [20] in a biomechanical study to significantly decrease graft lengthening in cyclic loading but had no effect on ultimate graft failure. 

We routinely pretensioned all our grafts and readjusted loop tension after several flexion-extension cycles in our series, resulting in only one failure in a patient due to excessive preoperative mobility, who should have received, in hindsight, additional fixation possibly via ALL reconstruction. 

Similarly, tunnel widening although originally a concern, has been shown to occur less often in short tunnel button fixation than interference screw fixation [21]. In fact, Ohori et al. [22] found that the length of the tendon in the tunnel was proportional to tunnel widening in a series of BPTB graft ACL reconstructions. It seems, therefore, that it is the unsupported tendon length that can freely move in the tunnel and not the fixation method that is the main factor leading to tibial tunnel widening. 

Overall, all-inside ACL reconstruction has been shown to have equivalent outcomes to classic S-G and BPTB reconstruction at mid-term follow-up [6,11,17,18,23]. Even in the more demanding younger patients, where the failure rates have been higher [24] the all-inside technique has wielded favorable outcomes [25]. There is, however, a paucity of studies with longer follow-up, possibly due to the novelty of the technique.

Given the equivalence in failure rates, some of the benefits of the less-invasive nature of the technique have caused an increased usage among surgeons. Donor site morbidity has been shown to be reduced both in terms of anterior knee pain compared to BPTB graft [25,26] and overall VAS pain scores compared to S-G harvest [11]. Kouloumentas et al. [23] reported improved knee flexion strength at two years following a single semitendinosus graft compared to S-G grafts. We also found improved VAS pain scores in the all-inside group compared to the full tibial tunnel S-G group, but the difference was significant only in the early (six months) postoperative period.

Other potential benefits include graft versatility [1,11,26], the use of the technique in skeletally immature patients due to the shorter tunnels required [5], and the use of the technique as a potential bailout when encountering short or inadequate S-G tendons [27].

The main limitations of the current study are the relatively small number of patients enrolled, which does not allow for analysis of potential confounding factors such as meniscal status and chondral damage, and the length of follow up, which at a minimum of two years does not cover the five-year mark, timepoint when most failures seem to have happened by [24].

## Conclusions

The all-inside technique with dual suspensory fixation and quadrupled semitendinosus graft appears to be equivalent to the classic interference screw technique with a semitendinosus-gracilis graft in terms of outcomes and failure rates.

The less-invasive nature of the technique with single tendon harvest and short tibial and femoral tunnels allows for improved early outcomes and does not burn any bridges for possible future revision surgery. In addition, it allows for greater graft versatility and can serve as a bailout plan in case short tendons are encountered or physeal-sparing procedures are required.
